# Evaluation of the probiotic, technological, safety attributes, and GABA-producing capacity of microorganisms isolated from Iranian milk kefir beverages

**DOI:** 10.3389/fmicb.2024.1385301

**Published:** 2024-06-05

**Authors:** Minoo Moghimani, Helen Onyeaka, Mohammad Hashemi, Asma Afshari

**Affiliations:** ^1^Department of Nutrition, Faculty of Medicine, Mashhad University of Medical Sciences, Mashhad, Iran; ^2^School of Chemical Engineering, University of Birmingham, Edgbaston, United Kingdom; ^3^Medical Toxicology Research Center, Mashhad University of Medical Sciences, Mashhad, Iran

**Keywords:** kefir, food microbiology, probiotic potential, gastrointestinal tract, gamma-aminobutyric acid, thin-layer chromatography, safety characteristics, *Enterococcus faecalis*

## Abstract

**Introduction:**

Kefir beverage has beneficial microorganisms that have health-giving properties; therefore, they have a good potential to be probiotic. This study evaluated the probiotic potential, technological, and safety characteristics of *Enterococcus faecalis*, *Lactococcus lactis*, and *Pichia fermentans* isolated from traditional kefir beverages.

**Method:**

First, isolates were evaluated in terms of resistance to acid, alkali, bile salts, trypsin, and pepsin of the gastrointestinal tract. The auto-aggregation and co-aggregation ability of isolates were measured using spectrophotometry. Antimicrobial activities were assayed against important food-borne pathogens using the agar well diffusion method. Moreover, gamma-aminobutyric acid (GABA) production was investigated by thin-layer chromatography (TLC).

**Result:**

Among the isolates, *P. fermentans* had an 85% total survival rate, but its amount reached below 6 log CFU/ml which is considered non-resistant, and it showed the highest auto-aggregation (74.67%). Moreover, only *L. lactis* showed antimicrobial activity and had the highest co-aggregation with *E. coli* PTCC 1338 (54.33%) and *L. monocytogenes* ATCC 7644 (78%). Finally, an evaluation of the technological and safety characteristics of the strains showed that the strains produced GABA and were safe.

**Discussion:**

Although the isolates were not resistant to the gastrointestinal tract, their supernatant contained valuable natural compounds, including antioxidants, GABA, and antimicrobials, which can be used to produce functional foods and medicines. In addition, other approaches, such as increasing the initial number of strains, using foods as carriers of isolates, and encapsulating the isolates, can effectively increase the survivability of isolates in the gastrointestinal tract.

## 1 Introduction

Although in the past healthy humans were just considered safe sources of probiotics, since FAO and WHO announced that the function of probiotics is more important than their source, scientists’ attention has been drawn to functional foods as new sources of probiotics (Guo et al., 2011; Guetouache and Guessas, 2015; LeBlanc et al., 2020; Bs et al., 2021; Bangotra et al., 2023). Among the functional foods, dairy-fermented products are consumed more, and the demand for them is higher (Nielsen et al., 2014). Kefir which is one of these products is a low-alcohol, viscous, and easily digestible carbonated beverage obtained by fermenting milk. Microorganisms that inhabit kefir grains, an insoluble protein and polysaccharide matrix, carry out the fermentation (Nielsen et al., 2014; Guetouache and Guessas, 2015; Mitra and Ghosh, 2020; Azizi et al., 2021; Touranlou et al., 2023).

A large number of these microorganisms have various merits, such as improving the immune system, preventing the growth of pathogenic microorganisms, antioxidant activity, hypocholesterolemic effect, controlling plasma glucose, antihypertensive, improving digestion, reducing the effects of obesity, reducing heart hypertrophy, and kidney hypertrophy (by producing vitamins, short-chain fatty acids, and bioactive substances like antioxidants, and gamma-aminobutyric acid) and prevent disease (by producing antimicrobial compounds) (Leite et al., 2015; Rosa et al., 2017; Mantzourani et al., 2019; Ganatsios et al., 2021; Moghimani et al., 2023). Therefore, kefir microorganisms are suitable candidates for being probiotic (Guetouache and Guessas, 2015; Gul et al., 2018; LeBlanc et al., 2020; Bs et al., 2021; Bangotra et al., 2023). In addition, Previous studies showed that the characteristics of microorganisms can be strain-dependent, so a strain-by-strain assessment of probiotic potential, health benefits, and safety of microorganisms is necessary (Leite et al., 2015; Bangotra et al., 2023; Erfani et al., 2023; Sionek et al., 2023; Zamanpour et al., 2023).

Probiotics have health-giving effects on the host when they reach the small intestine as live and active cells, for this reason, they must be resistant to the acidic and alkaline pH of the stomach, bile salts, pepsin, and pancreatin enzymes. Moreover, there are other factors besides resistance to stomach pH and bile salts to evaluate the probiotic potential, including the auto-aggregation ability for colonization in the intestine, co-aggregation ability with pathogens, and antimicrobial activity to inhibit the pathogens (Barzegar et al., 2021; Doğan and Ay, 2021; Goktas et al., 2021; Almeida et al., 2022; He et al., 2022).

Since probiotics are classified as Generally Recognized as Safe (GRAS) and Qualified Presumption of Safety (QPS) compounds, they must be checked for safety, especially *Enterococcus*, which is known as an opportunistic pathogen (Zendeboodi et al., 2020; Ozma et al., 2021).

Probiotics’ technological properties can be assessed to aid in their industrial application. Technological characteristics include the production of bioactive and beneficial compounds that increase cell survival rates (Zendeboodi et al., 2020; Barzegar et al., 2021). Gamma-aminobutyric acid (GABA) is one of these compounds. In the central nervous system, GABA, a four-carbon non-protein amino acid, functions as an inhibitory neurotransmitter. GABA has positive effects, such as treating insomnia, suppressing depression, improving long-term memory, and regulating blood pressure in the brain. Between the synthetic and biological methods of GABA production, biological production has received more attention due to its higher efficiency, lower cost, and environmental risks. A large group of microorganisms, including lactic acid bacteria and yeasts, can biologically produce GABA (Ribeiro et al., 2018; Ly et al., 2019; Perpetuini et al., 2020; Bs et al., 2021; Falah et al., 2021; Khanlari et al., 2021; Ghafurian Nasab et al., 2022).

Among the articles that assessed the probiotic potential of kefir’s microorganisms in various geographical regions, just Rahmani et al. (2022) assessed the probiotic potential of Iranian kefir beverage’s yeasts. This study isolated different species of yeast including *Saccharomyces cerevisiae*, *Kluyveromyces marxianus*, *Pichia fermentans*, and *Pichia kudriavzevii* that showed one strain of *P. fermentans* and three strains of *S. cerevisiae* are proper candidates as probiotic yeast (Rahmani et al., 2022). Other studies were related to Argentina, Korea, Brazil, Turkey, Malaysia, Singapore, and Mexico (Carasi et al., 2014; Leite et al., 2015; Zanirati et al., 2015; Cassanego et al., 2017; Cho et al., 2018; Azhar and Munaim, 2019; Talib et al., 2019; Yerlikaya, 2019; Akpinar and Yerlikaya, 2021; Doğan and Ay, 2021; Hurtado-Romero et al., 2021; Tan et al., 2022; Youn et al., 2022).

Hurtado-Romero et al. (2021) and Tan et al. (2022) were the only studies that examined the ability of kefir’s microorganisms to produce GABA. Hurtado-Romero et al. (2021) reported that *Lactococcus. lactis* (BIOTEC006, BIOTEC007, BIOTEC008), *Kluyveromyces. lactis* (BIOTEC009), *Leuconostoc. pseudomesenteroides* (BIOTEC012), and *Lentilactobacillus. kefiri* (BIOTEC014) isolated from Mexican kefir beverage were able to produce GABA (Hurtado-Romero et al., 2021). Tan et al. (2022) reported that just *Lentilactobacillus hilgardii* (Kef-w8, Kef-w9, Kef-w10) isolated from Singapore kefir had GABA synthetic genes (Tan et al., 2022).

In general, studies revealed that the microorganisms isolated from kefir beverages in different geographical regions are various and have a great potential to be probiotic (Carasi et al., 2014; Zanirati et al., 2015; Cassanego et al., 2017; Englerová et al., 2017; Cho et al., 2018; Bengoa et al., 2019; Talib et al., 2019; Akpinar and Yerlikaya, 2021). Therefore, the present study aims to assess the probiotic potential, biochemical and technological properties, and the safety of two bacterial species *Lactococcus lactis*, and *Enterococcus faecalis*, and a yeast species *Pichia fermentans* isolated from traditional Iranian kefir beverage.

## 2 Method

### 2.1 Study design

**Figure 1:**
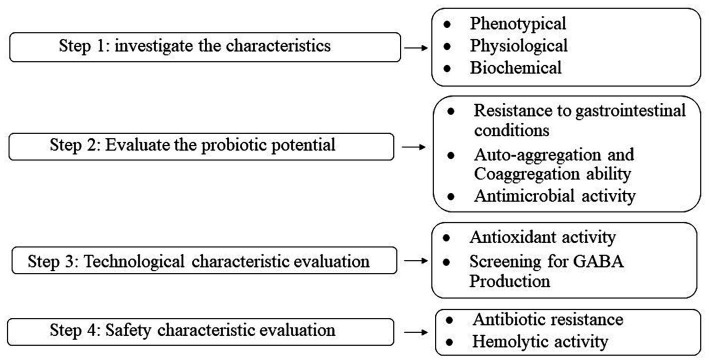
Flowchart of the experimental procedures in the present study.

A schematic flow chart of the experimental procedures used to investigate the characteristics of microorganisms and evaluate the probiotic potential, technical, and safety characteristics is shown in Figure 1.

### 2.2 Isolation and identification

*Enterococcus faecalis* (Accession number PP790751), *Lactococcus lactis*, (Accession number PP826201) and *Pichia fermentans* (Accession number PP803455) were isolated and identified from Iranian milk kefir beverages in our previous study using polymerase chain reaction (PCR) (Moghimani et al., 2023).

### 2.3 Examination of phenotypic, biochemical, and physiological characteristics

#### 2.3.1 Phenotypic characteristics

The morphology of colonies was examined based on colony shape, color, edge, size, bacterial cell shape, and Arrangement.

#### 2.3.2 Biochemical and physiological characteristics

The study examined the fermentation patterns of various sugars, specifically mannitol, glucose, lactose, sucrose, and xylose, in different bacterial strains. Additionally, the bacteria were analyzed using Gram staining. For yeast, lactophenol cotton blue staining was employed. The study also included a catalase test for enzyme activity and assessed the bacteria’s ability to grow at a temperature of 45°C.

### 2.4 Probiotic potential

The probiotic potential of the isolates was evaluated by examining four common tests, including resistance to the gastrointestinal tract, auto-aggregation ability, co-aggregation ability with pathogens, and antimicrobial activity.

#### 2.4.1 Resistance to the gastrointestinal tract

##### 2.4.1.1 Resistance to different pH, bile salts, simulated gastric, and intestinal juice

###### 2.4.1.1.1 Preparation of isolate samples

Overnight cultures were spun at 6,000 rpm for 15 min. The supernatant was discarded, and the remaining cell pellets were washed twice with phosphate-buffered saline (PBS) at a pH of 7.2. The concentration of these cell pellets was adjusted to 1.5 × 10^8^ CFU/mL.

###### 2.4.1.1.2 pH resistance test

One milliliter of the prepared isolates was mixed with 9 mL of PBS adjusted to different pH levels: 2.5 (simulating gastric conditions), 8 (simulating intestinal conditions), and 7 (control). These mixtures were incubated at 37°C for 3 h. The survival of cells at 0 and 3 h was assessed by cultivation on de man–rogosa–sharpe agar (MRS) and potato dextrose agar (PDA) (Ibresco) plates (Baccouri et al., 2019).

###### 2.4.1.1.3 Bile salt resistance test

For testing resistance to bile salts, 1 mL of isolates at a concentration of 1.5 × 10^8^ CFU/mL was combined with 9 mL of MRS (Condalab) and yeast extract peptone dextrose (YPD) (Quelab) broth containing 0.3% bile salts (Sigma-Aldrich). These were incubated at 37°C for 4 h, with cell survival analyzed at 0 and 4 h using MRS and PDA agar plates. Broths without bile salts served as controls (Baccouri et al., 2019).

###### 2.4.1.1.4 Simulated digestive juice test

To mimic gastric juice, a solution containing 3 g/L of pepsin (Sigma-Aldrich) at pH 2.5 was prepared. For intestinal juice, a solution containing 0.15% bile salts and 0.1% pancreatin (Sigma-Aldrich) at pH 8 was used. Each isolate was first exposed to gastric juice for 3 h, centrifuged, washed with PBS, and then exposed to intestinal juice for another 3 h at 37°C. Cell survival was evaluated at 0 and 3 h post-exposure to each juice type (Barzegar et al., 2021; Afshari et al., 2022).

Results were put in the following equation to obtain the percentage of survival rates.


$$\text{Survival}\;\text{Rate}\;\left(\%\right)=\frac{\log\;CFU\;N1\;}{\log\;CFU\;N0}\times 100$$

N_1_ = The number counted in the final time.

N_0_ = The number counted at time 0.

#### 2.4.2 Auto-aggregation and co-aggregation ability

The overnight culture of isolates was centrifuged at 6,000 rpm for 15 min. Their supernatant was discarded and the pellets were washed twice with PBS at a pH of 7.2. Isolates with the concentration of 1.5 × 10^8^ CFU/mL were vortexed for 10 s and incubated at 37°C for 24 h. To obtain the auto-aggregation percentage, the absorbance of isolates was measured by a spectrophotometer (Jenway, England) at 600 nm in 0, 2, 4, 6, 8, and 24 h (*Lactobacillus casei* PTTC 1608 was used as standard probiotic strain). Finally, the percentage of auto-aggregation was determined according to the following equation (Barzegar et al., 2021).


$$\text{Auto}-\text{aggregation}\;\left(\%\right)=\frac{A0-A1\;}{A1}\times 100$$

A_0_ = Absorption at 0 h.

A_1_ = Absorption at 2, 4, 6, 8, and 24 h.

To evaluate the co-aggregation ability, an equal amount of isolates and pathogenic bacteria, including *Escherichia coli* (PTCC 1338) and *Listeria monocytogenes* (ATCC 7644) with the concentration of 1.5 × 10^8^ CFU/mL were prepared, mixed, and vortexed for 10 s. The absorbance of the mixture suspensions was measured at 600 nm at 0, 2, 4, 6, 8, and 24 h by a spectrophotometer (*Lactobacillus casei* PTTC 1608 was used as the standard probiotic strain). The percentage of Co-aggregation was calculated according to the following equation:


$$Co-\text{aggregation}\;\left(\%\right)=\frac{\left(\frac{\text{AX}+AY}{2}-A\left(X+Y\right)\right)}{\frac{\text{AX}+AY}{2}}\times 100$$

*A_X_*: Absorbance of each isolate at 0 h.

*A_Y_*: Absorbance of pathogen 0 h.

*A*_(*X* + *Y*)_: Absorbance of the mixture suspension at 2, 4, 6, 8, and 24 h.

#### 2.4.3 Antimicrobial activity

The study assessed the antimicrobial properties of certain isolates using the agar well diffusion method on agar plates. This test was conducted against four types of bacteria: *Listeria monocytogenes* (ATCC 7644), *Bacillus cereus* (ATCC 14579), *Salmonella Typhimurium* (ATCC 14028), and *Escherichia coli* (PTCC 1338). Initially, 1.5 × 10^8^ CFU/mL of each bacterial strain was spread on Muller Hinton agar (Condalab) plates. Subsequently, wells of 6 mm diameter were created in the agar. The cell-free supernatant (CFS) of the isolates was prepared by centrifuging their overnight cultures at 6,000 rpm for 15 min, followed by filtration through a 0.22 μm filter. 100 μL of this supernatant was then added to each well. The plates were incubated at 37°C for 24 h, with sterile distilled water serving as blank (Almeida et al., 2022).

### 2.5 Technological properties

#### 2.5.1 Antioxidant activity

The antioxidant activity of the isolates was measured using a DPPH (1-diphenyl-2-picrylhydrazyl) assay. For this test, an equal volume of each isolate’s CFS was mixed with 1.5 mL of ethanolic DPPH (Sigma-Aldrich) solution (0.4 mmol). This mixture was incubated at 37°C in the dark for 1 h. The absorbance of the solution was then measured at 517 nm. The control for this test was a mixture of 1.5 mL DPPH and 1.5 mL methanol. Antioxidant activity, expressed as scavenging activity percentage, was calculated using the following formula (Archer and Halami, 2015; Sakkaa et al., 2022).


$$\text{Scavenging}\;\text{activity}\;\left(\%\right)=\frac{\left(A\;\text{control}-A\;\text{sample}\right)\;}{A\;\text{control}}\times 100$$

#### 2.5.2 Screening for GABA production

The ability of the isolates to produce GABA (Gamma-Aminobutyric Acid) from monosodium glutamate (MSG) was determined using thin-layer chromatography (TLC). The isolates were grown in MRS and YDP broth with 1% MSG at 37°C for 48 h. Then, 2 μL of their CFS was applied on a silica gel TLC plate (60 F256, Sigma-Aldrich). The applied spots were positioned 2 cm from the bottom and 1 cm apart from each other and the plate edges. GABA and MSG were also applied as controls. The plate was exposed to a mobile phase consisting of butanol, acetic acid, and distilled water (5:2:2, v/v/v), and removed once the solvent front reached two-thirds of the plate height. The plate was then sprayed with a ninhydrin solution and heated at 105°C for 5 min. The retention factor (Rf) for each spot was calculated, and isolates showing the same Rf as the GABA standard were identified as GABA producers (Falah et al., 2021; Ghafurian Nasab et al., 2022; Sakkaa et al., 2022).


$$\text{Retention}\;\text{factor}=\frac{\text{Distance}\;\text{traveled}\;by\;\text{spot}}{\text{Distance}\;\text{traveled}\;by\;\text{the}\;\text{solvent}}$$

### 2.6 Safety evaluation

#### 2.6.1 Antibiotic resistance

The antibiotic resistance of the isolates was assessed using the disk diffusion method. For this purpose, overnight cultures of the isolates, at a concentration of 1.5 × 10^8^ CFU/mL, were spread on MRS and PDA agar plates. Antibiotic disks, including penicillin, ampicillin (10 mg per disk), erythromycin (15 mg per disk), vancomycin, chloramphenicol, and tetracycline (30 mg per disk), along with a filter paper disk as a control, were placed on the agar, ensuring they were spaced apart. The plates were then incubated at 37°C for 24 h. The diameters of the inhibition zones around each disk (ZDI values) were measured and interpreted according to the Clinical and Laboratory Standards Institute (CLSI) 2009 guidelines. The results were categorized as follows: resistant (ZDI: ≤ 15 mm), sensitive (ZDI: ≥ 21 mm), or intermediately susceptible (ZDI: 16–20 mm) (Katiku et al., 2022).

#### 2.6.2 Hemolytic activity

To evaluate the hemolytic activity of the isolates, their overnight culture was cultured on blood agar plates (supplemented with 7% human blood), and the plates were incubated at 37°C for 48 h (Lakhlifi et al., 2023).

### 2.7 Statistical analysis

All data are shown as mean ± standard deviation of three independent replicates. Statistical data analysis was performed using Microsoft Excel 2016 and SPSS 16 with independent t-test, pair t-test, and one-way ANOVA followed by Tukey’s test.

## 3 Results and discussion

### 3.1 Phenotypic, biochemical, and physiological characteristics

**Figure 2:**
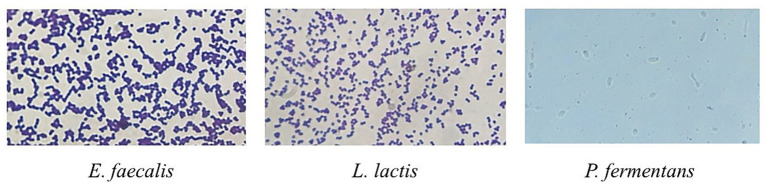
Microscopic image of Gram staining of *E. faecalis* and *L. lactis* strains and lactophenol cotton blue staining of *P. fermentans.* Bacteria were imaged at an original magnification of 1,000× and the yeast was imaged at the original magnification of 400×.

The results are reported in Table 1. Microscopic images of microorganisms are also shown in Figure 2.

**Table 1:** Morphological, biochemical, and physiological characteristics of the isolates.

				*E. faecalis*	*L. lactis*	*P. fermentans*
Characterization	Phenotypical	Colony morphology (macroscopically)	Shape	Circle	Circle	Circle
			Color	Beige	White	Milky White
			Edge	Smooth	Smooth	Undulate
	Biochemical	Cell morphology (microscopically)	Shape	Cocobacill	Cocobacill	oval
			Size (mm)	1–2	2–3	3–4
			Arrangement	Single-pair-chain	Single-pair-chain	Single
		Carbohydrate fermentation	Mannitol	Positive	Positive	–
			Glucose	Positive	Positive	–
			Lactose	Positive	Positive	–
			Sucrose	Positive	Negative	–
			Xylose	Negative	Negative	–
	Physiological	Gram staining		Positive	Positive	–
		Catalase		Negative	Negative	Positive
		Growth at 45°C		Positive	Negative	–

With regard to phenotypic, biochemical, and physiological characteristics of the isolates, results of the present study were consistent with previous studies, but there were some differences in sugar fermentation patterns which is because of the difference between studied strains (Leite et al, 2015; Hejazi et al., 2019; Hurtado-Romero et al, 2021). For example, Hejazi et al. (2019), reported that *E. faecalis* isolated from cheese was unable to ferment saccharose, while *E. faecalis* in the present study fermented it (Hejazi et al., 2019).

### 3.2 Probiotic potential

#### 3.2.1 Resistance to the gastrointestinal tract

##### 3.2.1.1 Resistance to different pH, bile salts, and simulated gastric and intestinal juice

Since an important principle about the effectiveness of probiotics is that they have to reach the target organ—the large intestine—alive and reproducible, they must be able to cope with the high acidity and alkaline pH, bile salts as well as gastric and intestinal juice in the digestive tract (Barzegar et al., 2021).

The resistance of isolates to different pH (2.5, 8, and 7), bile salts, and gastric and intestinal juice are shown in Tables 2–5, respectively. According to the results, *E. faecalis* and *L. lactis* could not tolerate the harsh conditions of the digestive tract, while *P. fermentans* tolerated these conditions with a total survival rate of 85%.

**Table 2:** Survival ability of the isolates in pH = 2.5, 8, and 7.

Isolates	Number of colonies in CFU/ml								
	pH = 2.5			pH = 8			pH =7		
	0 h	3 h	SR (%)	0 h	3 h	SR (%)	0 h	3 h	SR (%)
*E. faecalis*	7.39 ± 0.05	0	0	7.17 ± 0.14	7.17 ± 0.14	100	7.39 ± 0.28	7.18 ± 0.05	97
*L. lactis*	6.38 ± 0.03	0	0	7.31 ± 0.09	6.90 ± 0.21	94	7.15 ± 0.13	7.14 ± 0.08	99
*P. fermentans*	7.11 ± 0.06	7.15 ± 0.02	100	7.30	7.30	100	7.07 ± 0.17	7.17	101

The results were reported as mean ± standard deviation in three replicates. The number zero means not tolerating the applied conditions and not being able to grow and survive in those conditions.

**Table 3:** The resistance of the isolates to 0.3% bile salt.

Isolates	Number of colonies in CFU/ml				SR (%)	
	Time 0		Time 4			
	Sample	Control	Sample	Control	Sample	Control
*E. faecalis*	7.01 ± 0.14	7.88 ± 0.13	6.81 ± 0.06	7.68 ± 0.04	97	97
*L. lactis*	6.57 ± 0.06	7.83 ± 0.17	0	7.54 ± 0.11	0	96
*P. fermentans*	7.24 ± 0.01	7.24 ± 0.1	7.89 ± 0.21	7.92 ± 0.08	108	109

The results were reported as mean ± standard deviation in three replicates. The number zero means not tolerating the applied conditions and not being able to grow and survive in those conditions.

**Table 4:** The resistance of the isolates to simulated gastric juice.

Isolates	Number of colonies in CFU/ml				SR (%)	
	Time 0		Time 3			
	Sample	Control	Sample	Control	Sample	Control
*E. faecalis*	6.88 ± 0.1	7 ± 0.09	0	7 ± 0.09	97	97
*L. lactis*	6.32 ± 0.03	7.21 ± 0.24	0	7.18 ± 0.17	0	96
*P. fermentans*	6.14 ± 0.04	6.31 ± 0.01	5.83 ± 0.15	6.12 ± 0.07	108	109

The results were reported as mean ± standard deviation in three replicates. The number zero means not tolerating the applied conditions and not being able to grow and survive in those conditions.

**Table 5:** The resistance of the isolates to simulated intestinal juice.

Isolates	Number of colonies in CFU/ml				SR (%)	
	Time 0		Time 3			
	Sample	Control	Sample	Control	Sample	Control
*E. faecalis*	0	6.36 ± 0.02	0	6.33 ± 0.04	0	100
*L. lactis*	0	7.71 ± 0.24	0	7.3 ± 0.17	0	94
*P. fermentans*	5.44 ± 0.07	5.46	5.25 ± 0.07	5.32 ± 0.02	85	91

The results were reported as mean ± standard deviation in three replicates. The number zero means not tolerating the applied conditions and not being able to grow and survive in those conditions.

Multiple studies indicate that the ability of different bacterial isolates to withstand conditions in the gastrointestinal tract is influenced by several factors, including the acidity (pH), bile salt concentration, digestive enzymes (pepsin and pancreatin), incubation duration, and the specific strain of the bacteria (Leite et al., 2015; Baccouri et al., 2019; Merchán et al., 2020; Rahmani et al., 2022; Tan et al., 2022; Kanak et al., 2023). Our study specifically examined how isolates react to a pH level of 2.5 over a 3-h period, as these conditions closely resemble the average acidity and food retention time in the human stomach. However, other research, such as Leite et al. (2015), has found that certain strains like *L. lactis* from Brazilian kefir can endure a pH of 3 for up to 3 h and a bile salt concentration of 0.3% for an hour (Leite et al., 2015). Additionally, the final concentration of *P. fermentans* fell below 10^6^ CFU/mL, which is insufficient for probiotics to effectively benefit the host Therefore, it’s important to consider both the survival rate and final concentration of the bacteria.

Despite the fact that the isolates in our study could not withstand the aforementioned digestive conditions, it should be noted that their survival could be enhanced by using a food matrix like kefir, which is easily digested and does not remain in the stomach for long. Other potential solutions include encapsulation and increasing the initial quantity of the probiotics (Barzegar et al., 2021).

#### 3.2.2 Auto-aggregation and co-aggregation ability

Auto-aggregation refers to the potential of cells to assemble themselves, involving complex interactions with cell surface components or secreted factors. On the other hand, co-aggregation is when cells adhere to pathogens, aided by protein compounds on their surfaces. Both mechanisms serve as antimicrobial strategies: auto-aggregation prevents pathogen attachment, while co-aggregation exposes pathogens more effectively to probiotic antimicrobial agents like bacteriocins (Hurtado-Romero et al., 2021; Rahmani et al., 2022; Kanak et al., 2023).

**Figure 3:**
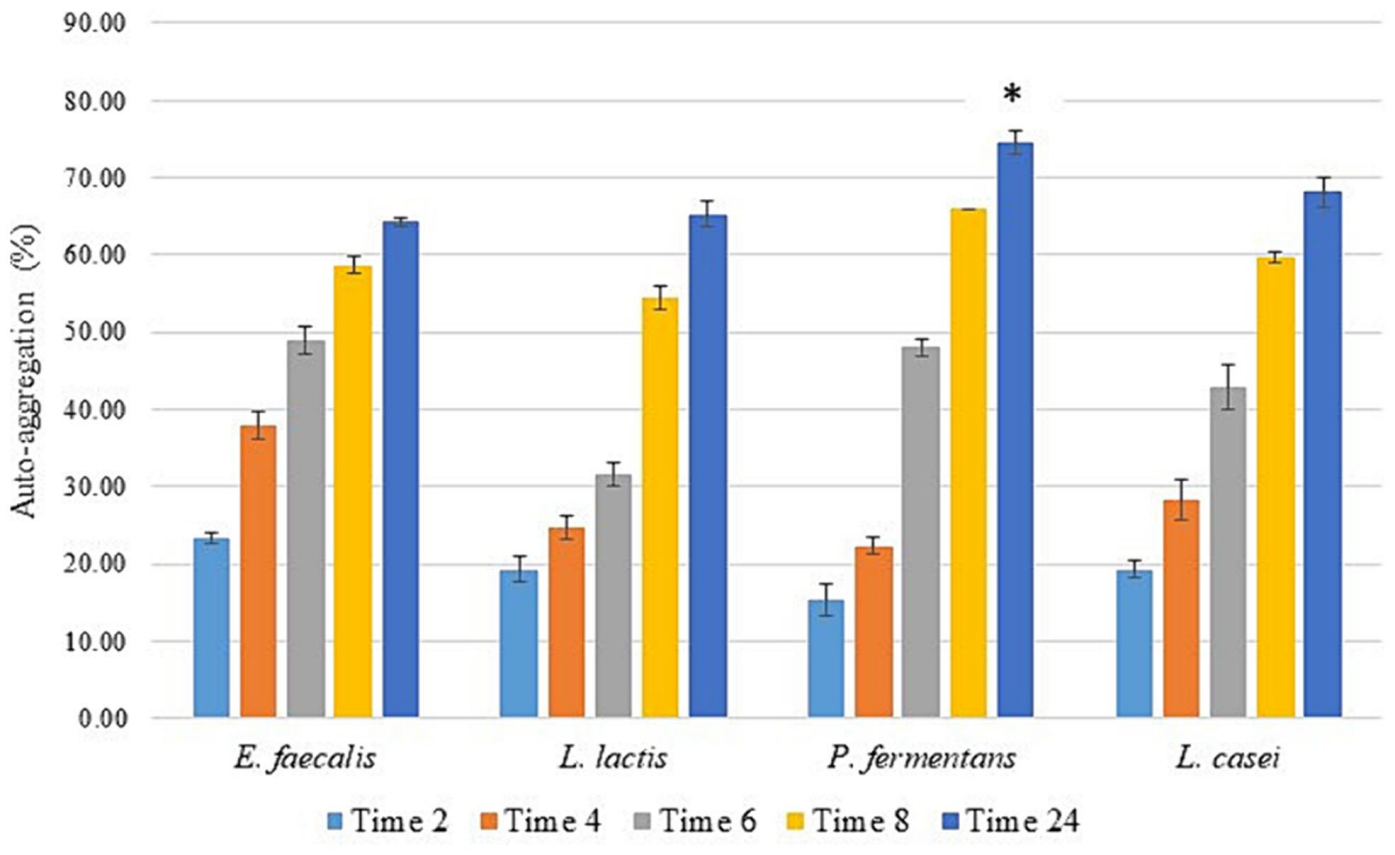
The percentage of auto-aggregation of the isolates as the average of three replicates with standard deviation at 2, 4, 6, 12, and 24 h of incubation at 37°C. *L. casei* was considered the standard probiotic strain. *indicates that *P. fermentans* had significantly the highest auto-aggregation activity among the strains and the standard strain after 24 h (*p*-value < 0.05).

As illustrated in Figure 3, all isolates exhibited a significant increase in auto-aggregation over time (*p-*value < 0.05), reaching 64–73% after 24 h. *P. fermentans* demonstrated the highest level of auto-aggregation, surpassing even the standard probiotic strain *L. casei* PTTC 1608, while *L. lactis* showed the lowest. However, the differences in auto-aggregation between *E. faecalis*, *L. lactis*, and the standard probiotic strain were not statistically significant (*p*-value > 0.05).

Various articles have demonstrated that the auto-aggregation of different probiotic strains is approximately 30–96% with an average of 62.6%, which increases over time. Therefore, the isolates of this study had a high percentage of auto-aggregation (Ogunremi et al., 2015; Baccouri et al., 2019; Kondrotiene et al., 2020; Merchán et al., 2020; Pytka et al., 2022; Rahmani et al., 2022; Youn et al., 2022; Kanak et al., 2023).

**Figure 4:**
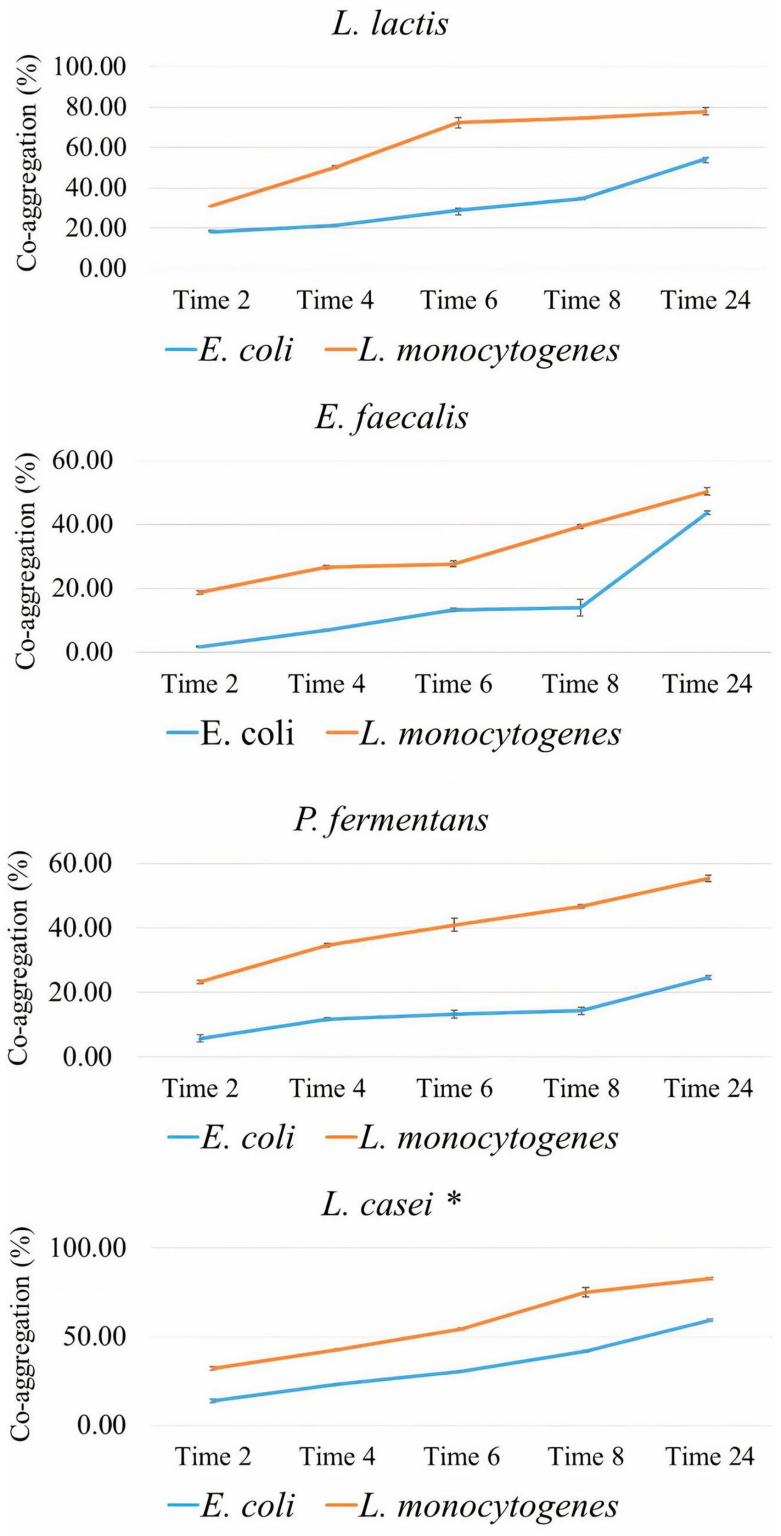
The percentage of co-aggregation of the isolates with *E. coli* and *L. monocytogenes* as the average of three replicates with standard deviation at 2, 4, 6, 12, and 24 h of incubation at 37°C. *L. casei* was considered the standard probiotic strain. *indicates that *L. casei* had significantly the highest co-aggregation activity with both food-borne pathogenes among the strains and the standard strain after 24 h (*p*-value < 0.05).

Figure 4 shows the co-aggregation percentage of the isolates with two food-borne pathogens including *E. coli* and *L. monocytogenes.* According to it, the co-aggregation percentage of all isolates and standard probiotic strain (*L. casei* PTTC 1608) with *L. monocytogenes* was significantly (*p*-value < 0.05) higher than *E. coli*. The co-aggregation of standard probiotic strain was significantly (*p*-value < 0.05) higher with both pathogens compared to all the isolates. The percentage of co-aggregation with both pathogens for the isolates increased significantly over time (*p*-value < 0.05).

However, the present study demonstrated that the percentage of co-aggregation of all isolates with Gram-positive pathogen (*L. monocytogenes*) was significantly higher than with Gram-negative pathogen (*E. coli*). Results of previous studies did not show a relationship between the percentage of co-aggregation and Gram stain of the pathogen (Nami et al., 2019; Pytka et al., 2022; Kanak et al., 2023; Yang et al., 2023). Research evidence has shown that the co-aggregation percentage is dependent only on incubation time, probiotics, and pathogen strain (Kanak et al., 2023).

#### 3.2.3 Antimicrobial activity

The antimicrobial property of probiotics is due to their ability to produce compounds such as organic acids (especially lactic and acetic acids), polyamines, proteases, and bacteriocins (Rahmani et al., 2022).

**Figure 5:**
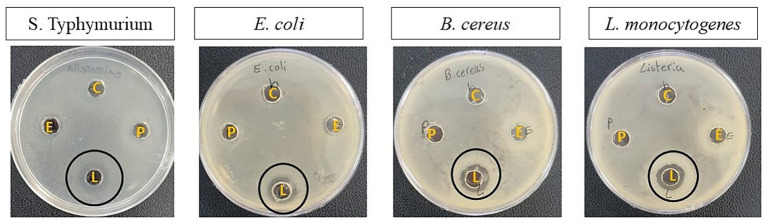
The antimicrobial activity of the isolates against *S. typhimurium*, *E. coli*, *B. cereus*, and *L. monocytogenes.* The letters B, E, L, and P represent blank (Sterile distilled water), *E. faecalis*, *L. lactis*, and *P. fermentans*, respectively.

Table 6 and Figure 5 show the antimicrobial activity of the isolates. According to them, *E. faecalis* and *P. fermentans* had no inhibitory effect while *L. lactis* had an antimicrobial effect on all pathogens studied. Among the pathogens, *L. lactis* had the most inhibitory effect on *L. monocytogenes* (*p*-value < 0.05) and its inhibitory effect on the other pathogens was not significantly different (*p*-value > 0.05).

**Table 6:** The diameter of the inhibition zone of the isolates against the pathogens of *L. monocytogenes*, *B. cereus*, *S. typhimurium*, and *E. coli.*

Isolates	Pathogenes			
	*L. monocytogenes*	*B. cereus*	*S. typhimurium*	*E. coli*
*E. faecalis*	0	0	0	0
*L. lactis*	12.66 ± 0.47	12 ± 0.81	18.33 ± 2.35	13.33 ± 1.24
*P. fermentans*	0	0	0	0

All values are in millimeters and are expressed as mean ± standard deviation. The number zero means the absence of an inhibition zone around the well.

Studies show that the antimicrobial property of probiotics is very different even in the same species and it depends on probiotics’ by-products and pathogen strains (Merchán et al., 2020; Hurtado-Romero et al., 2021; Rahmani et al., 2022; Tan et al., 2022). For instance, the results of the present study were not consistent with Hurtado-Romero et al. (2021)’s findings because *L. lactis* strains isolated from Brazilian kefir had no antimicrobial activity against the *E. coli*, *S. typhi*, and *S. aureus* (Hurtado-Romero et al., 2021).

The findings of the present study were similar to Rahmani et al. (2022) who reported *P. fermentans* strains isolated from Iranian kefir did not have any antimicrobial effect on *S. enterica*, *E. coli*, *E. faecalis*, *S. aureus*, and *Pseudomonas aeruginosa*. Merchán et al. (2020) also showed that *P. fermentans* strains isolated from cheese had no or very weak antimicrobial effect on the studied pathogens (Merchán et al., 2020; Rahmani et al., 2022).

Although scientific evidence has shown that the inhibitory effect of yeast is less than lactic acid bacteria, those yeast strains that cannot produce antimicrobial metabolites can prevent pathogen growth through other abilities such as auto-aggregation and co-aggregation (Rahmani et al., 2022).

### 3.3 Technological properties

#### 3.3.1 Antioxidant activity

Probiotics have the ability to release bioactive substances with antioxidant qualities that shield the body from oxidative stress, a condition that is directly linked to a number of illnesses, including aging, Parkinson’s disease, diabetes, and cancer (Lakhlifi et al., 2023). The antioxidant activity of probiotics is strain-dependent and there are various methods to evaluate it. Using the DPPH free radical is one of the typical ways to assess the antioxidant activity of microorganisms. This method is based on DPPH reduction in methanol by taking hydrogen from an antioxidant to form DPPH-H (Ogunremi et al., 2015; Baccouri et al., 2019).

**Figure 6:**
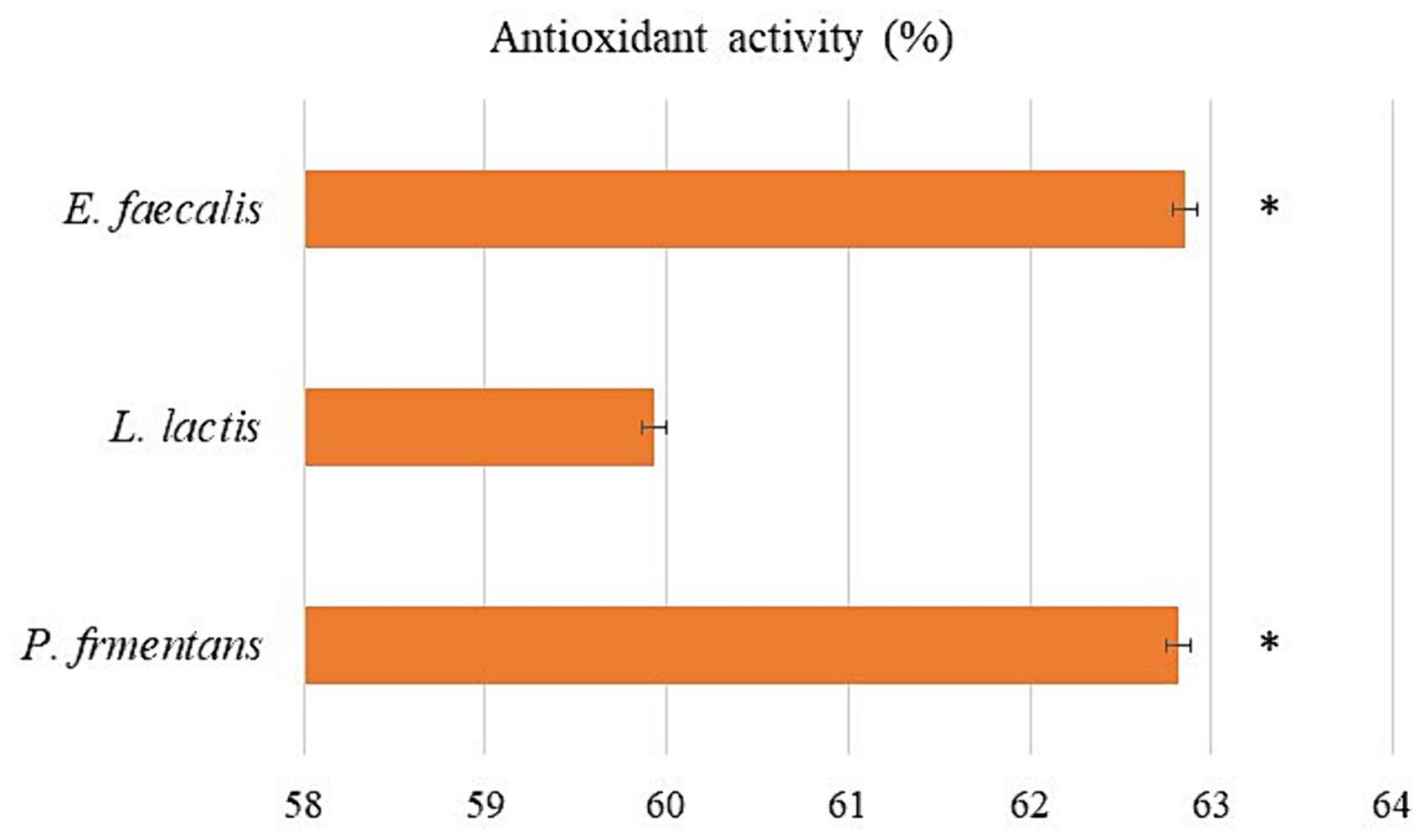
The antioxidant activity of the isolates as the average of three replicates with standard deviation. *indicates significance (*p*-value < 0.05).

As shown in Figure 6, the CFS of the isolates showed a great ability to scavenge DPPH. Among the isolates, the scavenging activity of *E. faecalis* and *P. fermentans* was significantly higher than *L. lactis* (*p*-value < 0.05).

Probiotic bacteria produce metabolites including glutathione, vitamins, and phenolic compounds such as carotenoids, which can prevent the production of free radicals or even destroy them, while the antioxidant activity of probiotic yeasts is mostly because of the presence of large amounts of beta-glucan in their cell walls (Amaretti et al., 2013; Ogunremi et al., 2015; Kotowicz et al., 2019; Hsu and Chou, 2021).

#### 3.3.2 Screening for GABA production

**Figure 7:**
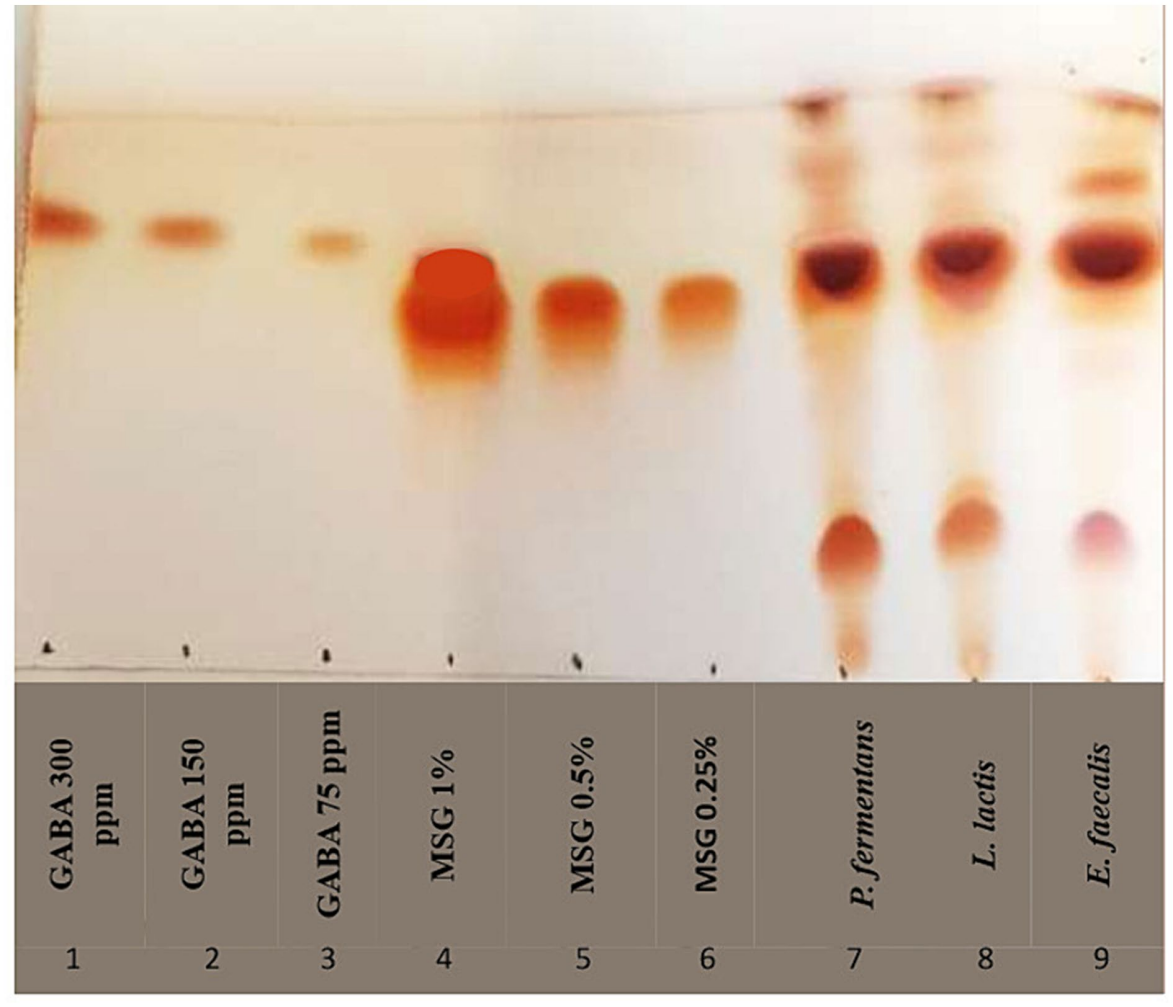
TLC chromatogram of GABA production of representative strains. As controls, lanes one to three and four to six contain varying quantities of gamma-aminobutyric acid (GABA) and monosodium glutamate (MSG), respectively; lanes seven through nine represent the isolates.

In this study, the GABA-producing potential of the isolates from MSG was investigated by TLC. The results in Figure 7 showed that the RF of all isolates was equal to the GABA standard RF (RF = 0.75) and the diameter of the spot for *E. faecalis, L. lactis*, and *P. fermentans* was 6, 7, and 9 mm, respectively, which qualitatively shows that *P. fermentans* had produced more GABA.

To the best of our knowledge, our study is the first study that has reported GABA production by *E. faecalis*. Franciosi et al. (2015) reported *E. faecalis* isolated from cheese was unable to produce GABA and the rest of the studies investigated other strains of *Enterococcus*, which demonstrated that *E. faecium* and *E. avium* were able to produce GABA (Tamura et al., 2010; Franciosi et al., 2015; Bs et al., 2021; Sakkaa et al., 2022). Moreover, *P. fermentans* was investigated for the first time in terms of GABA production in this study, while, previous studies showed that other *Pichia* species, including *P. Kudriavzevii*, *P. silvicola*, *P. Guilliermondii*, and *P. scolyti* had been able to produce GABA (Guo et al., 2011; Han and Lee, 2017; Li et al., 2022).

Psychobiotics are living bacteria that have directly and indirectly positive effects on the function of neurons by colonizing in the large intestine. Therefore, the production of GABA as a neurotransmitter is considered a psychobiotic property. Since GABA is regarded as a bioactive substance that supports health and is helpful for the development of foods for specified health uses (FOSHU), the food industry is primarily interested in its production especially by GABA-producing microorganisms because produce natural GABA (Martirosyan and Singh, 2015; Diez-Gutiérrez et al., 2020). For example, a germination technique was used by Cáceres et al. (2017) and Cho and Lim (2016) to increase the amount of GABA in brown rice, while El-Fattah et al. (2018) created functional yogurt that is high in bioactive compounds, including GABA (Cho and Lim, 2016; Cáceres et al., 2017; El-Fattah et al., 2018).

### 3.4 Safety evaluation

Since humans and animals consume probiotics, they should be safe and were assessed in this term. There are many doubts about the use of *Enterococci* bacteria as probiotics (Barzegar et al., 2021). Although *Enterococci* bacteria are not yet GRAS, in contrast to other LAB genera, and they are the main cause of nosocomial infections, previous studies have shown that some *Enterococci* bacteria such as *E. faecalis*, *E. faecium*, and *E. durans* have been approved as probiotics. For these reasons, before introducing a novel, potentially probiotic *Enterococcus* strain into functional food, its safety should be determined. A certain *Enterococcus* strain must be non-pathogenic, genetically stable, devoid of virulence and antibiotic resistance genes, particularly for vancomycine, in order to be considered safe. On the other hand, various *Enterococcus* species are part of the normal flora in the colon and their main pathogenicity is outside the digestive tract, therefore its oral consumption does not normally cause any problems (Iqbal et al., 2017; Baccouri et al., 2019; Nascimento et al., 2019; Nami et al., 2019; Kim et al., 2022; Sakkaa et al., 2022; Kanak et al., 2023).

#### 3.4.1 Antibiotic resistance

Although antibiotics are effective treatments for bacterial diseases, the indiscriminate use of broad-spectrum antibiotics has caused antibiotic resistance in some pathogens. The transmission of antibiotic-resistant genes by these pathogens in the food chain is very dangerous for human health. Therefore, probiotics should not be resistant to antibiotics (Azhar and Munaim, 2019; Hurtado-Romero et al., 2021; Kim et al., 2022).

**Figure 8:**
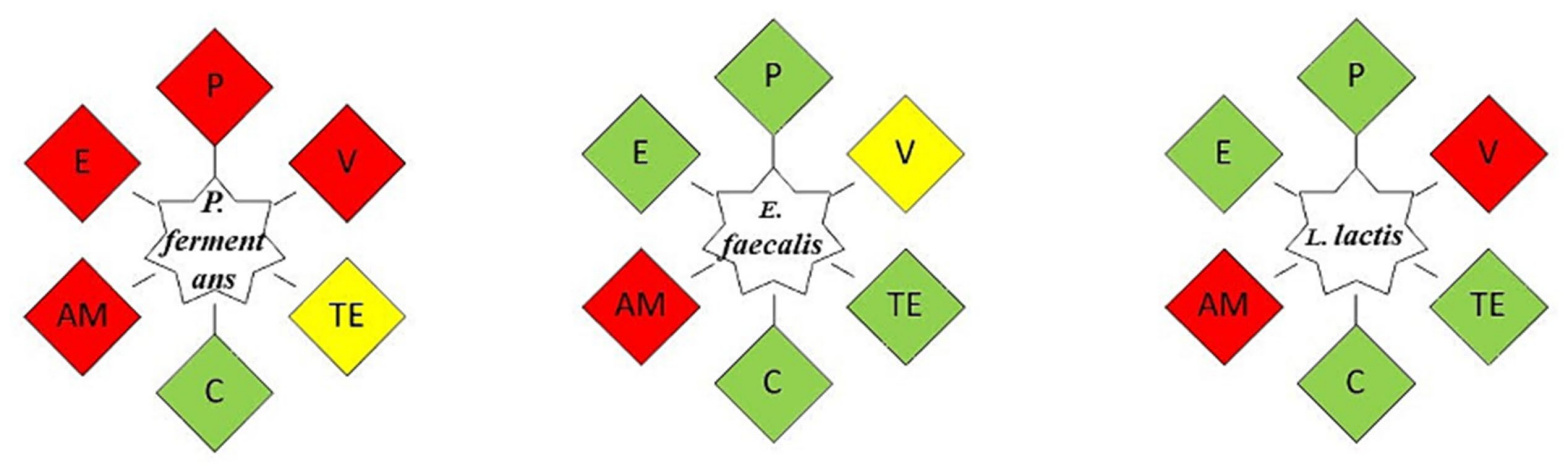
The antibiotic resistance of the isolates. As an indicator of the isolate’s susceptibility to the intended antibiotic, green represents its sensitivity to it, yellow indicates its relative resistance to it, and red shows its resistance. P, Penicillin; V, Vancomycin; TE, Tetracycline; C, Chloramphenicol; AM, Ampicillin; E, Erythromycin.

The antibiotic resistance results summarized in Figure 8 show that *E. faecalis* was resistant only to ampicillin and *L. lactis* to ampicillin and vancomycin, while the yeast strain was resistant to most antibiotics.

Since antibiotic resistance varies depending on the strain, many articles have revealed different results about it. Moreover, the source of antibiotic resistance genes is another factor that can influence antibiotic resistance; if it is intrinsic, it cannot be passed on, which is a quality that may be desired; More specifically, this property enables probiotics to restore the gut microbiota during or following antibiotic therapy; but, if it is acquired, it poses a risk of spreading to other microorganisms. Although the isolates in this investigation did not exhibit phenotypic resistance to the majority of antibiotics, it is crucial to look into the existence of antibiotic resistance genes in future research and, in the following phase, determine whether these genes are inherent or acquired (Azhar and Munaim, 2019; Hurtado-Romero et al., 2021).

#### 3.4.2 Hemolytic activity

Hemolysins are protein enzymes or non-protein toxins that cause cellular disruption; This mechanism involves creating pores in the cell membrane. There are three types of hemolysis generated by bacteria: alpha (α), beta (β), and gamma (γ). Alpha hemolysis is the relative lysis of red blood cells that results in the colony area turning green following incubation. Gamma hemolysis does not cause hemolysis. On the other hand, in β hemolysis, the red blood cells undergo complete lysing and following incubation, the colony turns transparent. Therefore probiotics must be hemolysin-free (Kim et al., 2022; Rahmani et al., 2022).

None of the isolates showed β-hemolysis after 24 h, which is consistent with the results of Baccouri et al. (2019), Kanak et al. (2023), Yang et al. (2023), and Rahmani et al. (2022).

## 4 Limitations

- Failure to investigate virulence factors, especially in the *E. faecalis* strain.

## 5 Conclusion

In the contemporary global landscape, there is a marked and increasing interest in the production and consumption of functional foods, attributed to their health benefits. This study delves into the realm of kefir, a widely acclaimed functional beverage, renowned for its unique properties. Research has consistently linked the therapeutic qualities of kefir to the diverse microorganisms present within kefir grains. Our investigation focused on analyzing microorganisms isolated from Iranian kefir, scrutinizing their probiotic potential, technological merits, and safety attributes.

While these isolates displayed limited resistance to the conditions of the digestive tract, they exhibited promising results in several other key areas. Notably, the Cell-Free Supernatant (CFS) of these isolates was found to contain antioxidant compounds and Gamma-Aminobutyric Acid (GABA), a compound of significant value. These components present exciting opportunities for the extraction and development of novel functional food products. Furthermore, the CFS of *Lactococcus lactis* demonstrated a potent inhibitory effect on four common food-borne pathogens, highlighting its potential as a natural antimicrobial agent. This is particularly relevant given the current high demand for such natural compounds in the food industry.

Considering the limited digestive tract resistance of these isolates, the study proposes two strategic approaches to enhance their efficacy. First, the use of encapsulation techniques involving biomaterials could offer better protection to the probiotics to tackle the harsh conditions of the digestive tract. Secondly, the development of more robust and targeted delivery systems is suggested. Such systems could significantly improve the stability and survival rate of these microorganisms, ensuring that they retain their beneficial properties throughout the digestive process. This dual approach could be pivotal in maximizing the therapeutic potential of kefir-derived probiotics, thereby contributing to the broader field of functional food development.

## Data availability statement

The raw data supporting the conclusions of this article will be made available by the authors, without undue reservation.

## Author contributions

MM: Data curation, Formal analysis, Investigation, Methodology, Software, Writing – original draft. HO: Methodology, Writing – review & editing. MH: Methodology, Resources, Writing – review & editing. AA: Conceptualization, Funding acquisition, Project administration, Resources, Supervision, Validation, Visualization, Writing – review & editing.
