# Supplementary figures and images for: Evaluation of the probiotic, technological, safety attributes, and GABA-producing capacity of microorganisms isolated from Iranian milk kefir beverages
Source: Front Microbiol. 2024 Jun 5;15:1385301. doi: 10.3389/fmicb.2024.1385301 (PMC11188319; doi:10.3389/fmicb.2024.1385301)

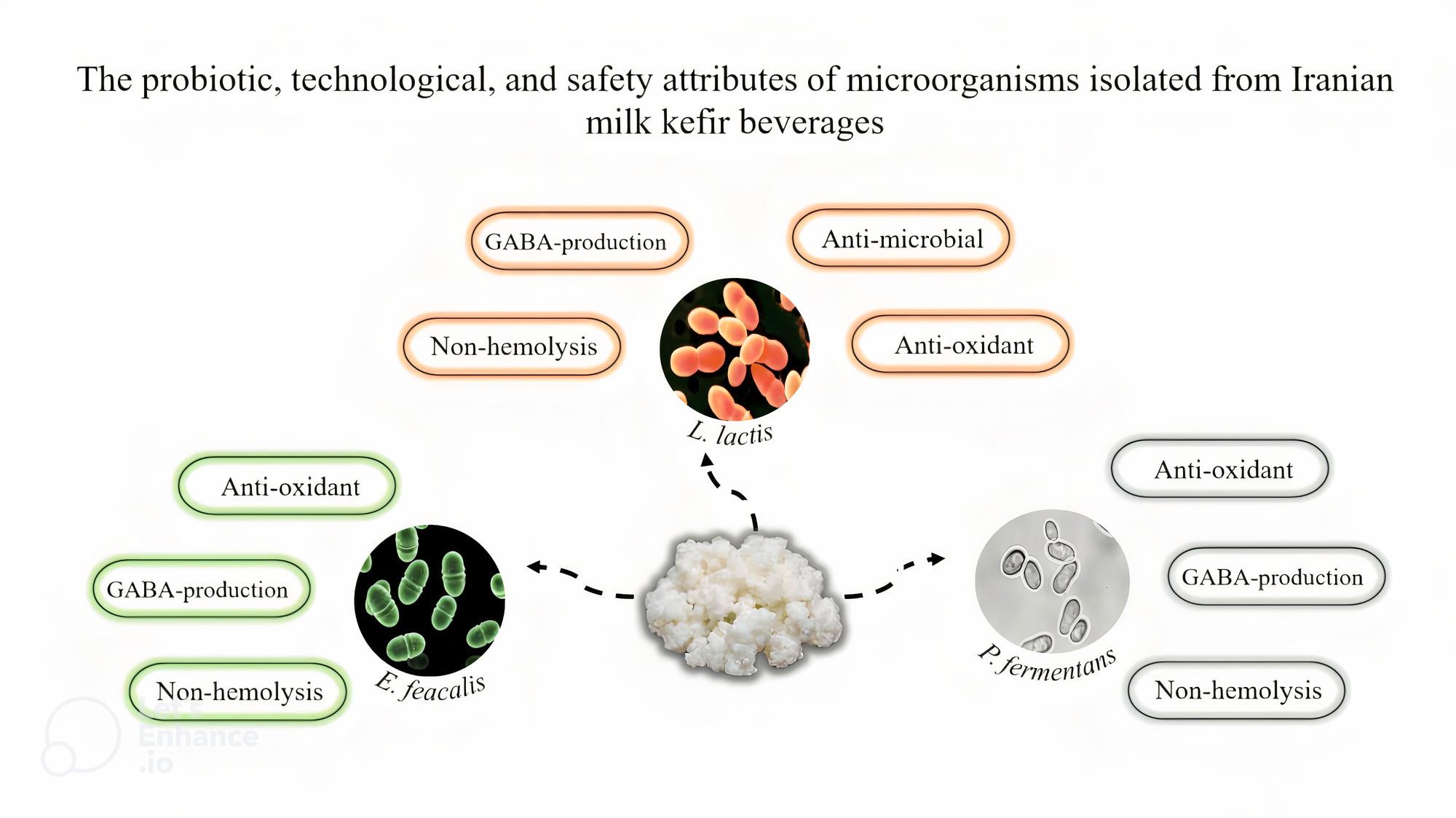

Supplement: Supplementary file 1 [file Image_1.jpg]
